# Preclinical Evaluation of Antiurolithiatic Activity of *Viburnum opulus* L. on Sodium Oxalate-Induced Urolithiasis Rat Model

**DOI:** 10.1155/2014/578103

**Published:** 2014-08-03

**Authors:** Mert İlhan, Burçin Ergene, Ipek Süntar, Serkan Özbilgin, Gülçin Saltan Çitoğlu, M. Ayşe Demirel, Hikmet Keleş, Levent Altun, Esra Küpeli Akkol

**Affiliations:** ^1^Department of Pharmacognosy, Faculty of Pharmacy, Gazi University, Etiler, 06330 Ankara, Turkey; ^2^Department of Pharmacognosy, Faculty of Pharmacy, Ankara University, 06100 Ankara, Turkey; ^3^Laboratory Animals Breeding and Experimental Researches Center, Faculty of Pharmacy, Gazi University, Etiler, 06330 Ankara, Turkey; ^4^Department of Pathology, Faculty of Veterinary Medicine, Afyon Kocatepe University, 03200 Afyonkarahisar, Turkey

## Abstract

The aim of the present research is to evaluate the antiurolithiatic effect of the various extracts prepared from the fruits of *Viburnum opulus* L., in regard to its ethnobotanical record. To induce urolithiasis, 70 mg/kg sodium oxalate was injected to the rats which were housed individually in metabolic cages. The test materials were applied during 7 days. Biochemical (urine and serum parameters), histopathological and antioxidant (TBARs, TSH and GSH) assays were conducted. The urine samples were examined by light microscope for the determination of the calcium oxalate crystals. Lyophilized juice of *V. opulus* (LJVO) and lyophilized commercial juice of *V. opulus* (LCJVO) exerted potential antiurolithiatic activity which was attributed to its diuretic effect along with the inhibitory action on the oxalate levels and free radical production. We also determined the chlorogenic acid content of the LJVO by high-performance liquid chromatography (HPLC). Chlorogenic acid was determined by using Supelcosil LC-18 (250 × 4.6 mm, 5 *µ*m) column and acetonitrile: water: 0.2% *o*-phosphoric acid as a mobile phase. The chlorogenic acid content of *V. opulus* was found to be 0.3227 mg/mL in fruit juice. The results obtained in this study have provided a scientific evidence for the traditional usage of *V. opulus* on passing kidney stones in Turkish folk medicine.

## 1. Introduction

Urolithiasis is a urinary stone disease involves the calcifications in the kidney, bladder, or urethra. Epidemiological studies have shown that the majority of stones are commonly composed of calcium oxalate (CaOx) [1]. The treatment of this disease includes medical and surgical treatment. However, surgical removal of stones by lithotripsy and percutaneous nephrolithotomy techniques can cause several side effects such as tubular necrosis, fibrosis of the kidney, and renal stone formation [2]. Medicinal plant materials could be beneficial to find out efficient cure for urinary stones [3]. Indeed, The World Health Organization has also paid importance to the use of herbal drugs as well as traditional medicines due to low cost and low side effects [4].

The genus* Viburnum* (Caprifoliaceae) is composed of more than 230 species distributed from South America to Southeast Asia, the majority of them being endemic. The genus is represented by four species in the flora of Turkey:* Viburnum opulus* L.,* V. orientale* Pallas,* V. lantana* L., and* V. tinus* L. [5].

The plant is known to contain iridoids, triterpenoids, diterpenoids, sesquiterpenes, coumarins, anthocyanins, phenolic acids, and organic acids [5, 6]. The biological activities of this plant could be related to these compounds. In our previous study, quantitative determination of chlorogenic acid and salisin in the leaves, branches, and fruits in* Viburnum opulus* and* V. lantana* was carried out by RP-HPLC using the external standard method. According to this study,* V. opulus* fruits are considered as a good chlorogenic acid source [7]. This genus was reported to possess antinociceptive [5], relaxant, spasmolytic [8], hepatoprotective, hypoglycemic [9], antioxidant [7, 10], antiacetylcholinesterase [10], and anticarcinogenic potential [11].

The fruits of* V. opulus* have been consumed as food and applied as folk remedy in Europe and Asia. The fruits have been reported to be utilized for the treatment of cough and cold, shortness of breath, high blood pressure, tuberculosis, stomach pain, digestive disorders, duodenal ulcers, kidney and bladder infections, and bleedings [12]. In Turkish folk medicine, the juice obtained by squeezing of the fruits of* V. opulus* is taken orally to pass kidney stones [13]. In inner Anatolia, a traditional drink named* gilaburu* has been prepared from the fruits of* V. opulus* [6]. The objective of the present study is to assess the antiurolithiatic effect of the extracts prepared from the fruits of* V. opulus,* in regard to its ethnobotanical record.

## 2. Materials and Methods

### 2.1. Plant Material

The fruits of* Viburnum opulus* were collected in 2012 from the vicinities of Kayseri, Turkey.

### 2.2. Preparation of the Extracts

Air-dried and powdered fruits of* V. opulus* were extracted sequentially with *n*-hexane, ethyl acetate (EtOAc), and methanol (MeOH). The extracts were prepared by macerating 50 g of powdered fruit in 300 mL of *n*-hexane and methanol for 8 hours. The macerates were evaporated until dryness. The juice of* V. opulus* was lyophilized.

### 2.3. High-Performance Liquid Chromatography (HPLC) Analysis

Chlorogenic acid content of the lyophilized juice of* V. opulus* (LJVO) was determined by using HP-1200 (Agilent Technologies Inc., California, USA).

### 2.4. Preparation of Standard Solutions

The stock solution is prepared by dissolving 10 mg chlorogenic acid (Sigma-Aldrich, Germany) in 10 mL methanol. The standards in six concentrations of the standard (0.1, 0.2, 0.4, 0.5, 0.6, and 0.8 mg/mL) were diluted from stock solution. The calibration equation was obtained by using 7 peak areas of standard solutions.

### 2.5. Preparation of Test Solution

10 mg LJVO was dissolved in 10 mL methanol. This solution is filtered from 0.45 *μ*m filters and directly injected.

### 2.6. Chromatographic Conditions

See Table 1.

### 2.7. Biological Activity Tests

#### 2.7.1. Preparation of the Test Samples for Bioassay

The extracts and Cystone were suspended in gum acacia (2% w/v) solution at the dose of 100 mg/kg and 500 mg/kg, respectively. The samples were administered through oral route by using oral gavage.

#### 2.7.2. Animals

Male Wistar rats (200–250 g) were obtained from Laboratory of Experimental Animals, Kobay, Turkey, and maintained under standard conditions with 12/12-hour light-dark cycles and were fed standard pellet diet and water* ad libitum*. The experiments were conducted in accordance with the Guide for the Care and Use of Laboratory Animals considering the animal experiments and biodiversity rights and the experiment was approved by the Experimental Animal Ethics Committee of Gazi University (G.U. ET-14.002). A minimum of six rats was used in each group.

#### 2.7.3. Experimental Design

A total number of 48 animals were randomly divided into following 8 groups. Vehicle group received 1 mL/kg gum acacia solution (2%). 70 mg/kg sodium oxalate was injected to the NaOx group as well as to the rest of the group animals intraperitoneally. Experimental groups received 100 mg/kg doses of* n*-hexane, EtOAc, MeOH, LJVO, and lyophilized commercial juice of* V. opulus* (LCJVO) in gum acacia solution, respectively. Cystone group, serving as the reference group, received 500 mg/kg suspended in gum acacia solution. The test materials were applied during 7 days. Rats were placed individually in metabolic cages (Techniplast, Milan, Italy) for 24 h for urine collection. During the experiment, food and water were available in the cages.

#### 2.7.4. Urine Parameters

Animals were placed in separate metabolic cages for 24 h and total urinary volume was measured by using the measuring cylinder and reported in mL. Level of creatinine (Jaffe's method), uric acid (Uricase method), magnesium, and oxalate [14] were determined in the urine samples.

On day 7, parameters such as leukocyte, nitrite, urobilinogen, protein, pH, blood, specific gravity, ketone, bilirubin, and glucose in the urine samples were measured by using Mission Urinalysis Reagent Strips.

#### 2.7.5. Urine Microscopy

The urine samples were explored under light microscope (Leica) after centrifugation at 1000 rpm at 4°C for 15 minutes using centrifuge machine (Jouan-MR 1822, France).

#### 2.7.6. Serum Parameters

The blood was taken by cardiac puncture from each rat at the end of the experiment. For the evaluation of serum parameters, the serum was separated by centrifugation at 3000 rpm at 4°C for 15 minutes using centrifuge machine (Jouan-MR 1822, France). Serum levels of calcium, blood urea nitrogen, uric acid, and creatinine were evaluated with Roche Cobas C501 autoanalyzer diagnostic kits by using spectrometer; sodium, chloride, and potassium levels were measured with Roche Cobas C501 autoanalyzer diagnostic kits by using Ion selective electrode methods. Microalbumin levels were detected (Roche Cobas C501 autoanalyzer diagnostic test) by using immunoturbidimetric method. Magnesium levels were detected by using Perkin Elmer atomic absorption spectrometer. Oxalate levels were measured with Trinity Biotech diagnostic kit by using spectrometer.

#### 2.7.7. Histopathological Analyses

At the end of the experiment (on day 7) the animals were sacrificed by cervical dislocation of the neck. Both kidneys were removed and one kidney from each rat was placed in formalin solution (10%) for histopathological analyses. Tissue samples were embedded in paraffin, cut into 5 *μ* fine sections with a rotary microtome, stained with hematoxylin-eosine (HE), and examined under a light microscope (Nicon Eclipse Ci attached both polarizing attachment and Kameram Digital Image Analyse System).

### 2.8. Determination of Antioxidant Activity

#### 2.8.1. Lipid Peroxidation, Thiobarbituric Acid Reactive Substances (TBARs)

The tissues were homogenized and cytosolic fraction was obtained by a two-step centrifugation. Homogenate was transferred to a vial and mixed with 8.1% (w/v) sodium dodecyl sulfate solution, 20% acetic acid solution (adjusted to pH 3.5), and 0.8% (w/v) TBA solution and the final volume was adjusted to 4.0 mL with distilled water. Each vial was heated for 60 min and then cooled under tap water. Equal volumes of tissue blank or test sample and 10% TCA were mixed. The absorbance of the supernatant fraction was measured at 532 nm by using Beckman DU 650 spectrometer. TBA solution was replaced with distilled water for the control group. 1,1,3,3-Tetraethoxypropane was used as standard for calibration of the curve [15].

#### 2.8.2. Total Thiols (TSH) and Glutathione (GSH)

Tissues were homogenized in ethylenediaminetetraacetic acid disodium (EDTA-Na_2_). The homogenate was mixed with 0.2 M Tris buffer (pH 8.2) and Ellman's reagent for the determination of total-SH groups. The mixture was brought to 10.0 mL with absolute methanol. The reaction mixture was centrifuged just after the color was developed. The absorbance of supernatant was measured at 412 nm.

For the determination of GSH, homogenate was mixed with distilled water and 50% TCA. Tubes were centrifuged and supernatant was mixed with 0.4 M Tris buffer (pH 8.9) and Ellman's reagent. The absorbance was read at 412 nm [16].

### 2.9. Statistical Analysis

The results are expressed as mean ± SEM. Comparison between the groups was made by analysis of variance (ANOVA) followed by Dunnett's test.

## 3. Results

The urinary output of NaOx group rats was 11.75 ± 1.21 mL/24 h/rat on day 7. On the other hand, it was determined as 25.21 ± 0.19 mL/24 h/rat in the LJVO group. The LCJVO group and Cystone group were found to be 24.00 ± 1.28 mL/24 h/rat and 19.56 ± 1.53 mL/24 h/rat, respectively (Table 2). Seven days administration of NaOx (70 mg/kg i.p.) resulted in increased oxalate (2.15 ± 0.74 mg/dL), uric acid (2.97 ± 0.56 mg/dL), and creatinine (4.25 ± 0.17 mg/dL) excretion in NaOx group. In LJVO treated group reduction in oxalate (0.69 ± 0.10 mg/dL), uric acid (1.96 ± 0.95 mg/dL), and creatinine levels (2.95 ± 0.24 mg/dL) was detected. Similarly, a reduction in oxalate (0.61 ± 0.09 mg/dL), uric acid (1.79 ± 0.81 mg/dL), and creatinine (2.25 ± 0.19 mg/dL) levels was observed in LCJVO treated group. Cystone treated group also caused remission on the oxalate (0.59 ± 0.03 mg/dL), uric acid (1.63 ± 0.75 mg/dL), and creatinine levels (1.39 ± 0.10 mg/dL). Magnesium excretion decreased to 0.09 ± 0.02 mg/dL significantly by NaOx administration. However, treatment with LJVO, LCJVO, and Cystone prevented urinary loss of magnesium and restored it to normal value (Table 2).

Administration of NaOx did not significantly alter the output of urine compared to vehicle group. The treatment of LJVO, LCJVO, and Cystone caused significant increase in urine compared to control group. Administration of NaOx increased the pH of urine as compared to untreated group. Administration of LJVO and LCJVO did not change the pH of urine whereas Cystone caused a reduction in the pH of urine compared to vehicle group (Table 2).

A significant increase in kidney weight was observed in the NaOx group when compared to the vehicle group. Treatment with LJVO, LCJVO, and Cystone groups reduced kidney weight significantly when compared to NaOx group (Table 2).

Parameters such as leukocyte, nitrite, urobilinogen, protein, blood, specific gravity, ketone, bilirubin, and glucose were presented as shown in Table 3.

**Figure 1:**
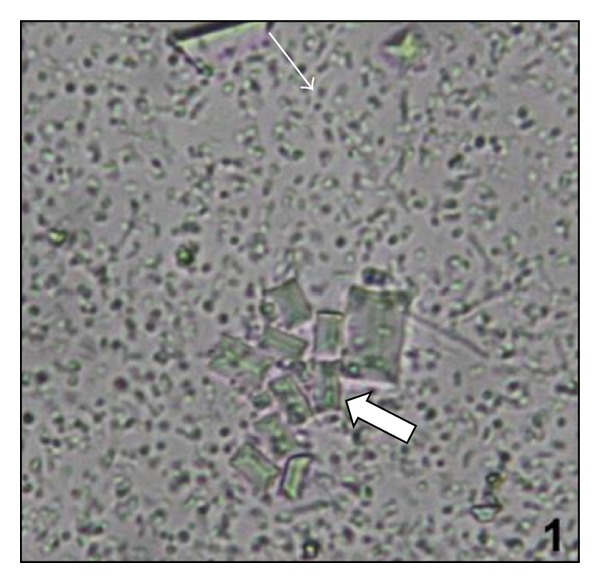
Microscopic view of urine samples (the original magnification is ×400). Data are representative of 6 animals per group; NaOx administered animals Arrow pointing the CaOx crystals.

**Figure 2:**
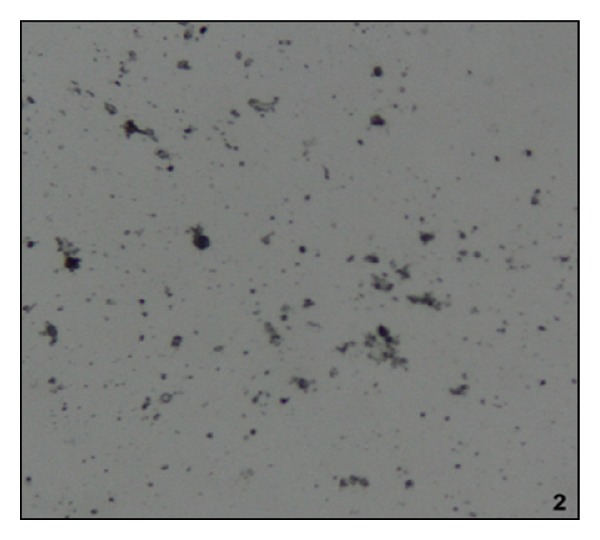
Microscopic view of urine samples (the original magnification is ×400). Data are representative of 6 animals per group; LJVO treated group.

**Figure 3:**
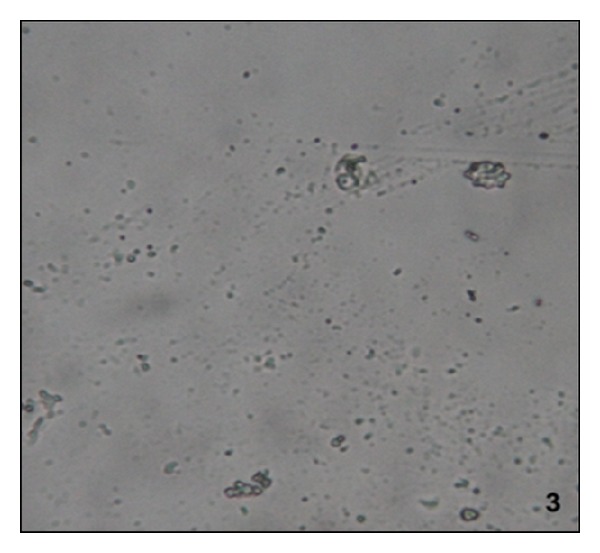
Microscopic view of urine samples (the original magnification is ×400). Data are representative of 6 animals per group; LCJVO treated group.

**Figure 4:**
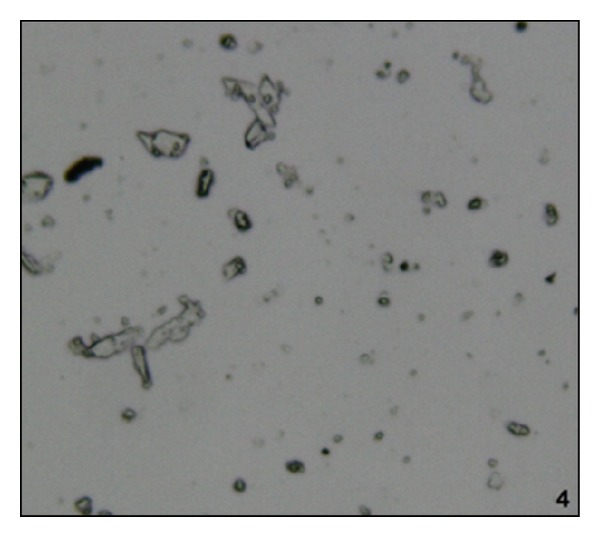
Microscopic view of urine samples (the original magnification is ×400). Data are representative of 6 animals per group; Cystone treated group.

Administration of NaOx caused significant increase in the urinary oxalate level. Maximum crystal deposition was detected in the urine sample of the NaOx group, while vehicle group and Cystone treated groups showed no crystal deposition. Urine samples of LJVO and LCJVO treated groups did not show any crystal deposition as well (Figures 1, 2, 3, and 4).

Increase in serum sodium, potassium, and uric acid levels was observed in NaOx group, which indicated impaired renal functions. Creatinine level also increased (4.06 ± 0.13 mg/dL) in NaOx group. On the other hand, creatinine levels were detected as 1.66 ± 0.10 mg/dL and 1.46 ± 0.09 mg/dL in LCJVO and Cystone group, respectively. The serum sodium, potassium, uric acid, and creatinine values significantly reduced in the LJVO, LCJVO, or Cystone treated groups compared to the NaOx group. The level of blood urea nitrogen was found to increase in the NaOx group. On the other hand, treatment with Cystone significantly lowered the increased level of blood urea nitrogen (Table 4).

**Figure 5:**
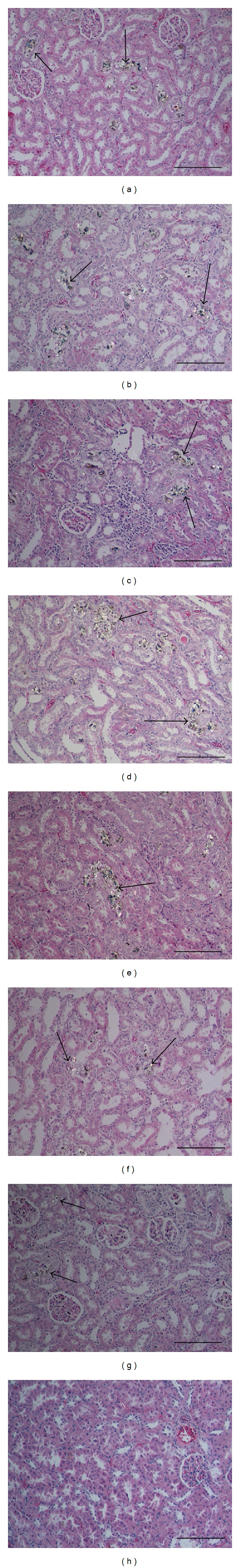
Histopathological view of the experimental groups. Sections show the hematoxylin and eosin (HE) stained kidney. Sections were viewed using a Nicon Eclipse Ci optical microscope and polarized light. Arrows indicates renal calculus. The original magnification was ×100 and the scale bars represent 150 *μ*m for figures in (a)–(h). (a) Vehicle; (b) NaOx; (c)* n*-hexane extract; (d) EtOAc extract; (e) MeOH extract; (f) LJVO; (g) LCJVO; (h) Cystone group.

During histopathological examination, varying amounts of calculi were seen in the kidneys of experimental animals. Irregularly shaped calculus fragments were mostly considered in the NaOx group and then in the* n*-hexane, EtOAc, MeOH, vehicle, LJVO, and LCJVO groups, respectively. No abnormal histopathology was seen in the reference group. There were a large number of intratubular hyaline cylinders in the EtOAc group. Interstitially located mononuclear inflammatory cells were mostly seen in the EtOAc group and lesser in the* n*-hexane and then the other groups. Interstitial fibrosis was detected very few in the LJVO and LCJVO groups, but increasing rates were seen in the NaOx,* n*-hexane, EtOAc, MeOH, and vehicle groups, respectively. Capsular inflammatory lesions were detected in the vehicle, MeOH, EtOAc, and NaOx groups. Hemorrhage and interstitial oedema were seen in the EtOAc group. Cystic tubular dilatation was mostly seen in the* n*-hexane, EtOAc, and MeOH groups and lesser in the other groups (Table 5 and Figure 5).

In the present study, the levels of TBARs, GSH, and TSH were analysed. In the NaOx group, increase in the level of TBARs and decrease in the level of GSH and TSH indicated impaired renal functions (Table 6). Therefore, it could be suggested that oxalate causes generation of free radicals and thus renders the renal tissue to loose its cell membrane integrity [17]. On the other hand, LJVO and LCJVO groups demonstrated antioxidant activity by exerting similar activity results with the reference group without any renal damage.

**Figure 6:**
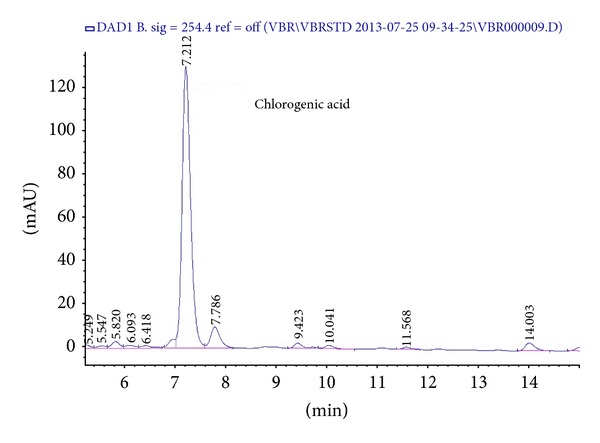
The HPLC chromatogram of LJVO.

**Figure 7:**
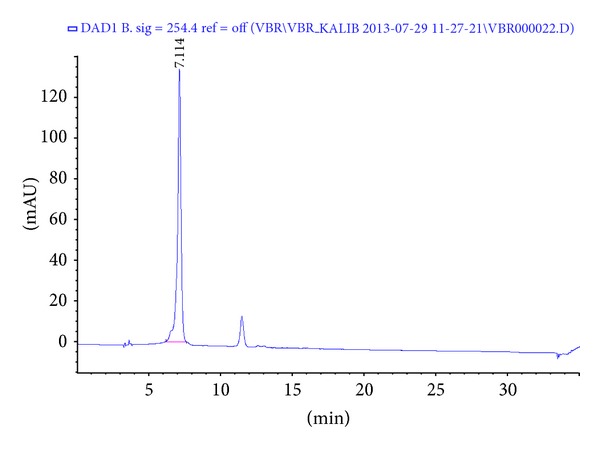
The HPLC chromatogram of chlorogenic acid.

As the result of quantitative analysis, the equation of chlorogenic acid was found to be *y* = 4602.1*x* − 28.643 (*r* ^2^ = 0.9997). The LOD (limits of detection) was calculated to be 0.2171 *μ*g/mL and the LOQ (limits of quantification) was calculated to be 0.7237 *μ*g/mL for chlorogenic acid. The chromatogram of LJVO and chlorogenic acid are shown in Figures 6 and 7, respectively. According to the equation, the chlorogenic acid content of LJVO is calculated as 3.227%.

## 4. Discussion

NaOx administration causes an increase in the severity of microscopic CaOx crystals deposition along with high crystal concentration in the kidney [17]. According to the ethnopharmacological data, fruit juice of* V. opulus* is used internally to pass kidney stones [12]. Therefore, in the present study, the activity of* V. opulus* extracts was investigated by using NaOx-induced urolithiasis model.

According to the urine microscopic examination, high amount of crystal deposition was detected in NaOx group, while no crystal deposition was detected in LJVO and LCJVO groups. Furthermore, accumulation of the CaOx crystals in the kidney increased the urinary pH, which is one of the indications of urolithiasis [18]. In the present study, the administration of NaOx increased the urine pH whereas the treatment of Cystone decreased. Coapplication of the extracts of* V. opulus* along with NaOx could probably decrease the pH of the urine which was increased due to the NaOx application. Therefore, the extract treated groups did not affect the pH of the urine. The components such as ascorbic acid, phenolic acid, and organic acid [19], found in* Viburnum* species, could probably balance the pH of the urine by their acidifier properties.

According to the results obtained herein, LJVO and LCJVO could be considered to have a potential diuretic activity. Indeed,* Viburnum* species were proven to have diuretic effect in the previous reports [19].

In the previous studies, in which the sodium oxalate-induced urolithiasis model was used, excessive urinary excretions of uric acid and creatinine have been detected [18]. Similarly herein, uric acid and creatinine excretion were found to be high in NaOx group, when compared to the LJVO, LCJVO, and the reference groups.

Low level of urinary magnesium is also an indication of the kidney stone [18]. Reduction in urinary magnesium level was detected in the NaOx group, while LJVO, LCJVO, and Cystone treatment improved the excretion of magnesium in the present study.

In urolithiasis, urea, creatinine, and uric acid levels in blood were found to be increased, due to the obstruction in the kidney, and decreased in urine output and glomerular filtration rate [18, 20]. In this context, the elevated levels of uric acid, urea nitrogen, and creatinine in serum were the indications of the renal damage [20]. The results of the serum parameters in the NaOx group were found to be much higher than those of the LJVO and LCJVO as well as Cystone group.

According to the histopathological findings, mainly renal calculus and also degeneration, cystic dilatations of tubules, intratubular hyaline cylinders, hemorrhage, and inflammation, urolithiatic lesions were listed from weak to severe as reference, LJVO and LCJVO, vehicle, MeOH, EtOAc, *n*-hexane, and NaOx groups, respectively.

Oxalate causes stone formation which induces lipid peroxidation and thus liver damage by reacting with unsaturated fatty acids in cell membranes [21]. In this study, increased level of TBARs was observed in NaOx administered group. However, elevation in lipid peroxidation was inhibited by the administration of the LJVO and LCJVO as well as Cystone. Moreover, GSH and TSH, which were known to decrease after renal function failure, significantly reduced in NaOx group. On the other hand, LJVO and LCJVO treated groups increased the levels of both GSH and TSH. In a recent research, six* V. opulus* genotypes were screened for their potential antioxidant activities [1]. The phenolic constituents of* V. opulus* were found to provide high antioxidant capacity [7]. In our previous studies the antioxidant activities of* V. opulus* and* V. lantana* were examined by using the 2,2-diphenyl-1-picrylhydrazyl (DPPH) scavenging and superoxide anion scavenging methods [22], ferrous ion chelating capacity, ferric reducing antioxidant power, and *β*-carotene bleaching assay [10]. Therefore, the antioxidant activity results of the present study were found to be in line with the previous data. On the basis of phytochemical researches,* Viburnum* species contain iridoids, triterpenoids, coumarins, flavones, tannins, anthocyanins, phenolic acids, and organic acids [7]. It is well known that the antioxidant activity of plant extracts is usually correlated with their phenolic contents. In this study, LJVO was analysed for their chlorogenic acid content (% 3.227) by HPLC. Phenolic compounds present in* V. opulus* may prevent the lipid peroxidation-induced renal damage caused by CaOx crystal deposition in the kidney [23]. Hypoglicemic, antihyperlipidemic, anti-inflammatory, and antioxidant activities of chlorogenic acid may improve the renal function in diabetic nephropathy [6, 24]. Antiurolithiatic activity of the LJVO and LCJVO could be related with the inhibition of oxalate levels and free radical production as well as diuretic activity which probably due to its phenolic contents.

## 5. Conclusion

The present study demonstrated the antiurolithiatic activity of the lyophilized juice of* V. opulus*. The promising results obtained in the present research have led to a scientific proof for the ethnopharmacological data on* V. opulus*.

## Figures and Tables

**Table 1:** 

Time (min)	Water (0.02% *o-*phosphoric acid) %	Acetonitrile %
0	94	6
25	70	30
30	70	30
30,01	0	100
35	0	100

Column: Supelcosil LC-18 (250 × 4.6 mm, 5 *μ*m).

Mobile phase: water (0.02% *o*-phosphoric acid) and acetonitrile were used in gradient elution.

Flow rate: 1 mL/min.

Detection: 254 nm (diode-array detector).

Injection volume: 10 *μ*L.

**Table 2:** Effect of the test samples on the urine parameters.

Groups	Unit	Statistical mean ± S.E.M.							
		Vehicle	NaOx	Hexane extract	EtOAc extract	MeOH extract	LJVO	LCJVO	Cystone
Creatinine	mg/dL	2.27 ± 0.09	4.25 ± 0.17	4.16 ± 0.09	3.97 ± 0.09	3.45 ± 0.27	2.95 ± 0.24∗	2.25 ± 0.19∗	1.39 ± 0.10∗∗
Urea nitrogen	mg/dL	18.42 ± 1.75	25.75 ± 0.98	28.59 ± 1.53	18.98 ± 1.43	21.14 ± 0.96	14.32 ± 1.17	13.23 ± 0.93∗	11.17 ± 0.84∗
Uric acid	mg/dL	0.37 ± 0.83	2.97 ± 0.56	2.95 ± 0.97	2.55 ± 1.16	2.06 ± 1.03	1.96 ± 0.95	1.79 ± 0.81	1.63 ± 0.75
Sodium	mEq/L	108.25 ± 3.76	128.51 ± 2.59	111.67 ± 2.09	100.25 ± 3.27	125.75 ± 2.19	107.12 ± 3.22	80.47 ± 4.56∗∗	73.85 ± 2.05∗∗∗
Potassium	mEq/L	72.54 ± 2.12	106.1 ± 2.76	126.3 ± 1.86	89.96 ± 2.36	97.71 ± 3.51	92.55 ± 3.02	88.20 ± 2.54	68.41 ± 1.92∗
Chloride	mEq/L	85.79 ± 2.66	149.15 ± 2.77	99.63 ± 2.81	116.25 ± 3.59	108.55 ± 3.27	96.43 ± 2.54	91.02 ± 1.69	82.45 ± 1.60
Calcium	mg/dL	4.88 ± 0.36	10.20 ± 0.21	8.61 ± 0.48	5.65 ± 0.55	5.82 ± 0.59	4.92 ± 0.29	5.02 ± 0.22	4.56 ± 0.18
Microalbumin	mg/L	2.41 ± 0.08	7.54 ± 0.07	2.55 ± 0.15	6.10 ± 0.36	4.99 ± 0.84	1.37 ± 0.23	1.02 ± 0.20∗	0.78 ± 0.18∗∗
Oxalate	mg/dL	1.54 ± 0.11	2.15 ± 0.74	1.99 ± 0.25	1.77 ± 0.21	1.02 ± 0.18	0.69 ± 0.10∗∗	0.61 ± 0.09∗∗	0.59 ± 0.03∗∗∗
Magnesium	mg/L	0.16 ± 0.05	0.09 ± 0.02	0.08 ± 0.06	0.11 ± 0.08	0.15 ± 0.10	0.10 ± 0.04	0.16 ± 0.07	0.15 ± 0.02
Volume of urine	mL	7.66 ± 1.15	11.75 ± 1.21	12.00 ± 1.85	8.00 ± 1.56	18.00 ± 1.44∗	25.21 ± 0.19∗∗	24.00 ± 1.28∗∗	19.56 ± 1.53∗
pH of urine	—	7.93 ± 0.14	9.00 ± 0.35	9.00 ± 0.31	8.75 ± 0.39	9.00 ± 0.27	7.92 ± 0.42	7.66 ± 0.42	7.25 ± 0.35
Kidney weight	g	1.22 ± 0.09	2.20 ± 0.05	1.92 ± 0.10	1.93 ± 0.08	1.95 ± 0.09	1.91 ± 0.06	1.89 ± 0.07	1.87 ± 0.08

S.E.M.: standard error of the mean; *P < 0.05; **P < 0.01; ***P < 0.001; test groups were compared to the vehicle.

**Table 3:** Effect of the test samples on the urine parameters.

Groups	Vehicle	NaOx	Hexane extract	EtOAc extract	MeOH extract	LJVO	LCJVO	Cystone
Leukocyte	15	70	15	70	15	15	15	15
Nitrite	0.7	—	—	—	—	—	—	—
Urobilinogen	0.2	0.2	0.2	0.2	0.2	0.2	0.2	0.2
Protein	1	2	2.6	2	1.5	1	—	—
Blood	5–10	50	5–10	1	5–10	1	1	—
Specific gravity	1005	1030	1010	1005	1000	1000	1000	1000
Ketone	—	—	—	—	—	—	—	—
Bilirubine	—	—	—	1	—	—	—	—
Glucose	—	—	—	—	—	—	—	—

**Table 4:** Effect of the test samples on the serum parameters.

Group	Unit	Statistical mean ± S.E.M.							
		Vehicle	NaOx	Hexane extract	EtOAc extract	MeOH extract	LJVO	LCJVO	Cystone
Creatinine	mg/dL	1.59 ± 0.04	4.06 ± 0.13	2.08 ± 0.12	2.26 ± 0.16	1.98 ± 0.07	1.75 ± 0.08	1.66 ± 0.10	1.46 ± 0.09
Urea nitrogen	mg/dL	11.17 ± 0.72	20.59 ± 1.32	18.89 ± 0.97	6.89 ± 1.07	9.15 ± 0.89	6.23 ± 0.70∗	5.02 ± 0.39∗∗	5.12 ± 0.45∗∗
Uric acid	mg/dL	1.99 ± 0.65	4.08 ± 0.15	2.05 ± 0.51	1.85 ± 0.05	2.19 ± 0.32	1.91 ± 0.40	1.88 ± 0.23	1.80 ± 0.16
Sodium	eEq/L	3213.42 ± 158.65	5144.76 ± 214.63	4123.48 ± 186.25	4486.32 ± 168.30	5026.42 ± 317.49	4073.29 ± 250.38	4215.82 ± 154.36	3961.03 ± 86.40
Potassium	eEq/L	32.62 ± 1.13	64.26 ± 1.08	44.31 ± 1.26	48.92 ± 1.32	50.76 ± 1.25	28.30 ± 1.02	30.54 ± 0.95	24.10 ± 0.80∗
Chloride	mEq/L	54.24 ± 2.75	132.73 ± 5.91	155.2 ± 3.67	81.96 ± 2.96	76.85 ± 3.04	74.17 ± 3.16	13.20 ± 1.91∗∗∗	10.35 ± 1.07∗∗∗
Calcium	mg/dL	2.74 ± 0.15	8.32 ± 0.39	7.14 ± 0.76	5.92 ± 0.50	3.85 ± 0.45	4.07 ± 0.36	2.53 ± 0.16	2.02 ± 0.09∗

S.E.M.: standard error of the mean; *P < 0.05; **P < 0.01; ***P < 0.001; test groups were compared to the vehicle.

**Table 5:** Urolithiasis and associated histopathologic lesions in the experimental groups.

Materials	Cf	Cdt	D	I	H	O	F	C	Hc
Vehicle	++/+++	++	+/++	+/++	−	−/+	+/++	+++	−
NaOx	+++	+/++	++	+	−	−/+	+	+/++	−
Hexane extract	++/+++	++/+++	++/+++	++	−	+	+	++	−/+
EtOAc extract	++/+++	++/+++	+++	++/+++	+	+	+	+/++	+/++
MeOH extract	++/+++	+++	++/+++	++/+++	−	−/+	+	++	−/+
LJVO	+/++	++	++	+/++	+	+	+	+++	−
LCJVO	+/++	+	+	+	−/+	−	−/+	++	−
Cystone	−	−	−	−	−	−	−	−	−

Cf: calculi fragments; Cdt: cystic dilatation of tubules; D: degeneration; I: inflammation; H: hemorrhage; O: oedema; F: fibrosis; C: capsulitis; Hc: hyaline cylinders.

**Table 6:** TBARS, GSH, and TSH levels in kidney.

Materials	Mean ± SD		
	TBARS	GSH	TSH
	(nmol/g)	(*μ*mol/g)	(*μ*mol/g)
Vehicle	266.2 ± 11.8	17.6 ± 1.4	7.7 ± 1.1
NaOx	258.8 ± 9.9	15.3 ± 0.9	7.1 ± 0.9
Hexane extract	207.3 ± 10.7	16.9 ± 1.2	5.4 ± 0.7
EtOAc extract	195.4 ± 10.4	15.9 ± 1.0	8.4 ± 1.3
MeOH extract	186.2 ± 8.4	19.9 ± 1.5	10.7 ± 1.2
LJVO	134.5 ± 6.5***	21.7 ± 1.2**	15.5 ± 1.7***
LCJVO	121.1 ± 5.1***	24.8 ± 1.1***	13.65 ± 1.9**
Cystone	125.5 ± 6.3***	25.2 ± 1.3***	17.4 ± 2.1***

**P < 0.01; ***P < 0.001.
